# SOX2-dependent expression of dihydroorotate dehydrogenase regulates oral squamous cell carcinoma cell proliferation

**DOI:** 10.1038/s41368-020-00109-x

**Published:** 2021-01-29

**Authors:** Xuemei Qiu, Sheng Jiang, Yanxuan Xiao, Yumin He, Tao Ren, Lu Jiang, Rui Liu, Qianming Chen

**Affiliations:** 1 State Key Laboratory of Oral Diseases & National Clinical Research Center for Oral Diseases & Chinese Academy of Medical Sciences Research Unit of Oral Carcinogenesis and Management, West China Hospital of Stomatology, Sichuan University, Chengdu, China; 2 Ministry of science and technology, The Second Affiliated Hospital of Chengdu Medical College (China National Nuclear Corporation 416 Hospital), Chengdu, China; 3 Oncology Department, Clinical Medical College and The First Affiliated Hospital of Chengdu Medical College, Chengdu, China

**Keywords:** Oral cancer, Metabolomics

## Abstract

Oral squamous cell carcinoma (OSCC) become a heavy burden of public health, with approximately 300 000 newly diagnosed cases and 145 000 deaths worldwide per year. Nucleotide metabolism fuel DNA replication and RNA synthesis, which is indispensable for cell proliferation. But how tumor cells orchestrate nucleotide metabolic enzymes to support their rapid growth is largely unknown. Here we show that expression of pyrimidine metabolic enzyme dihydroorotate dehydrogenase (DHODH) is upregulated in OSCC tissues, compared to non-cancerous adjacent tissues. Enhanced expression of DHODH is correlated with a shortened patient survival time. Inhibition of DHODH by either shRNA or selective inhibitors impairs proliferation of OSCC cells and growth of tumor xenograft. Further, loss of functional DHODH imped de novo pyrimidine synthesis, and disrupt mitochondrial respiration probably through destabilizing the MICOS complex. Mechanistic study shows that transcriptional factor SOX2 plays an important role in the upregulation of DHODH in OSCC. Our findings add to the knowledge of how cancer cells co-opt nucleotide metabolism to support their rapid growth, and thereby highlight DHODH as a potential prognostic and therapeutic target for OSCC treatment.

## Introduction

Head and neck cancer squamous cell carcinoma (HNSCC) ranks as the sixth-most common cancer worldwide, with an annual incidence of greater than 650 000 cases and causing 350 000 deaths annually.^1^ Oral squamous cell carcinoma (OSCC) is the most common type of HNSCC which refers to cancers that exist in the oral cavity, such as the mucosa of the lips, floor of the mouth, tongue, buccal mucosa, lower and upper gingival, hard palate, and retromolar trigone. Approximately 300 000 cases of OSCC occur every year, resulting in nearly 145 000 deaths worldwide.^2,3^ Development of OSCC is associated with multiple risk factors, among which tobacco use, alcohol consumption, and betel quid chewing are the most noteworthy. Other possible risk factors include oral microbiome, immune status, environmental pollutants, occupational exposures, heritable conditions.^2^ Though multiple therapeutic strategies, such as surgery, chemotherapy, radiotherapy, immunotherapy, or a combination of these modalities have been applied in OSCC treatment, lymph node metastasis and postoperative recurrence lead to a particularly poor prognosis and low 5-year survival rate.^3,4^ Therefore, identifying the key factors responsible for OSCC development and progression at molecular, genetic, and epigenetic levels is essential for improving the survival rate of OSCC patients.

**Fig. 1:**
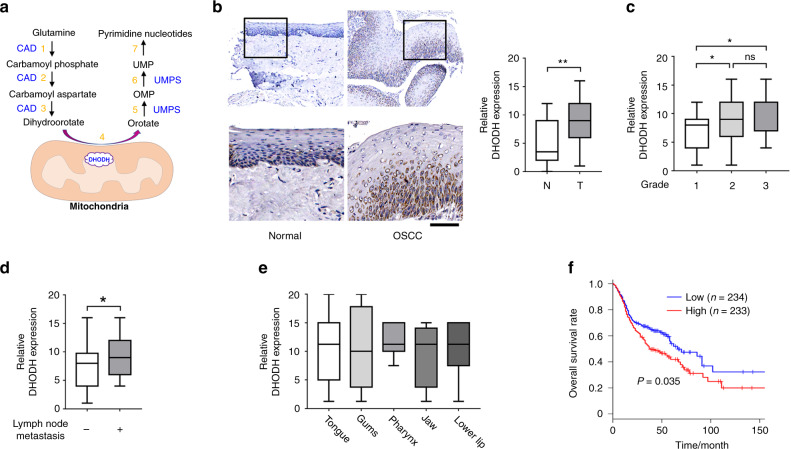
DHODH expression is upregulated in OSCC. **a** A schematic for de novo synthesis pathway of pyrimidine. Steps 1, 2, and 3 are catalyzed by Carbamoyl-Phosphate Synthetase 2, Aspartate Transcarbamylase, and Dihydroorotase (CAD); step 4 is catalyzed by DHODH; step 5 and 6 are catalyzed by Uridine Monophosphate Synthetase (UMPS). Step 7 indicates further reactions to generate UMP-derived pyrimidine nucleotides. **b** Expression of DHODH in OSCC tissues and non-cancerous adjacent tissues was examined by immunohistochemical staining. Representative staining images were shown (left panel). The boxed areas are enlarged. The staining intensities were scored and compared (right panel). **P < 0.01. Scale bar for original images, 250 μm; Scale bar for enlarged images, 100 μm. **c**–**e** Expression of DHODH in OSCC tissues with indicated classifications were compared. *P < 0.05. **f** DHODH expression was established by Kaplan–Meier method, and statistical comparison of these curves was performed by Log-rank test

Pyrimidines, including cytosine (C), thymine (T), and uracil (U), are essential building blocks for DNA replication and RNA transcription.^5^ The synthesis of pyrimidines is accomplished via two major pathways: de novo pyrimidine synthesis and salvage pathway. In mammalian cells, the de novo pyrimidine synthesis is the main source for producing pyrimidine nucleotides.^6^ CO_2_, glutamine, water, and aspartate are used as substrates to produce dihydroorotate and subsequently orotate. Orotate is then attached to the ribose-5-phosphate ring to form orotidine 5′-monophosphate (OMP), which is the precursor to generate cytosine, thymine, or uracil nucleotides (Fig. 1a).^7^ In this pathway, DHODH, a ubiquitously expressed mitochondrial inner membrane protein, governs the rate-limiting step by catalyzing the conversion of dihydroorotate to orotate through an oxidation reaction.^6,8,9^ It is widely established that nucleotide synthesis is frequently enhanced in cancer cells, to meet the increased demand for rapid cell proliferation.^10^ However, how DHODH is modulated in OSCC, and the role of DHODH in the regulation of OSCC cell proliferation remains elusive.

In this study, we demonstrate that the expression of DHODH was markedly increased in OSCC tissues, and was associated with multiple clinical parameters. Inhibition of DHODH by either shRNAs or selective antagonists reduced the proliferation rate and tumorigenicity of OSCC cell lines. Loss of DHODH dampens pyrimidine nucleotide synthesis and impairs mitochondrial homeostasis. Further, mechanistic study shows that the aberrant expression of DHODH in OSCC is regulated at transcriptional level by transcription factor SOX2.

## Results

### Expression of DHODH is upregulated in OSCC

We set out by examining the expression of DHODH in OSCC (*n* = 75) and non-cancerous adjacent oral mucous tissues (*n* = 30) through immunostaining. As shown in Fig. 1b, DHODH immunoreactivity was mainly observed in the perinuclear region, which was in line with previous reports showing mitochondrial localization of this protein.^11^ Notably, strong or moderate staining of DHODH was detected in tumor tissues; in contrast, most of non-cancerous tissues showed relatively weak DHODH signal (*P* < 0.001).

We further examined the correlation of DHODH with a set of clinicopathologic parameters. In contrast to those tumors diagnosed as Grade 1, DHODH staining was found to be more intense in the tumors of Grade 2 or 3 (Grade 2 vs. Grade 1, *P* = 0.018 1; Grade 3 vs. Grade 1, *P* = 0.012 9; Fig. 1c). No apparent differences in DHODH expression were found between Grade 2 or 3 tumors (Fig. 1c). Further, DHODH expression was substantially enhanced in OSCC patient with lymph node metastasis (*P* = 0.046 4, Fig. 1d). Nevertheless, no palpable correlation was observed between DHODH expression and tumor locations, including tongue, gums, pharynx, jaw, or lower lip (Fig. 1e).

To evaluate the impact of DHODH expression on the outcome of OSCC patients, we employed the online engine Gene Expression Profiling Interactive Analysis (GEPIA, http://gepia.cancer-pku.cn/detail.php) to analyzed 467 cases, whose gene expression profiling data is archived in The Cancer Genome Atlas (TCGA) or Genotype-Tissue Expression (GTEx). The survival curves of patients with high (n = 233) or low (n = 234) DHODH expression were established by Kaplan–Meier method, and statistical comparison of these curves was performed by Log-rank test. As results, those patients harboring a compromised DHODH expression showed a median survival time for approximately 67 months. While a largely shortened survival time was found for those patients with high DHODH expression (*P* = 0.035, Fig. 1f). These results strongly suggest that the expression of DHODH is upregulated in OSCC, and is corelated with the poor outcome of patients.

### Inhibition of DHODH impeded OSCC cell proliferation

**Fig. 2:**
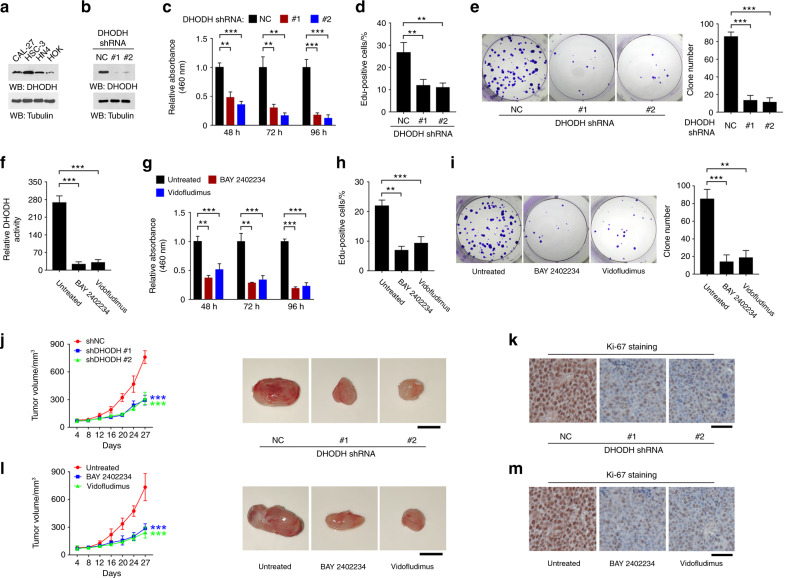
Inhibition of DHODH impairs OSCC cell proliferation. **a** Expression of DHODH in the indicated cell lines was examined by immunoblot. **b** HSC-3 cells were transfected with NC or DHODH shRNAs. 48 h after transfection, the expression of DHODH was examined by immunoblot. **c** HSC-3 cells were transfected with NC or DHODH shRNAs. 48, 72, or 96 h after transfection, cell viability was examined by CCK-8 assay. **P < 0.01; ***P < 0.001. **d** HSC-3 cells were transfected with NC or DHODH shRNAs. 72 h after transfection, cell proliferation was examined by EdU incorporation assay. **P < 0.01. **e** HSC-3 cells were transfected with NC or DHODH shRNAs. 72 h after transfection, cell proliferation was examined by colony formation assay. ***P < 0.001. **f** HSC-3 cells were treated with BAY 2402234 or Vidofludimus for 2 h, and DHODH activity in the cell lysate was examined. ***P < 0.001. **g** HSC-3 cells were treated with BAY 2402234 or Vidofludimus for 48, 72, or 96 h, and cell viability was examined by CCK-8 assay. **P < 0.01; ***P < 0.001. **h** HSC-3 cells were treated with BAY 2402234 or Vidofludimus for 72 h, and then cell proliferation was examined by EdU incorporation assay. **P < 0.01; ***P < 0.001. **i** HSC-3 cells were treated with BAY 2402234 or Vidofludimus for 72 h, and then cell proliferation was examined by colony formation assay. **P < 0.01; ***P < 0.001. **j**, **k** HSC-3 cells with stable expression of NC or DHODH shRNAs were subcutaneously injected in mice (*n* = 7). The tumor volume was recorded at indicated time (**j** left panel). Representative images of tumors were shown (**j**, right panel). Tumor cell proliferation was examined by Ki-67 assay (**k**). ***P < 0.001. Scale bar, 8 mm (**j**), 80 μm (**k**). **l**, **m** HSC-3 cells were subcutaneously injected in mice (*n* = 7). The tumor volume was recorded at the indicated time (**l**, left panel). Mice were treated with 5 mg/kg BAY 2402234 or 40 mg/kg Vidofludimus every other day during Day 8–16. Representative images of tumors were shown (**l**, right panel). Tumor cell proliferation was examined by Ki-67 assay (**m**). ***P < 0.001. Scale bar, 8 mm (**l**), 80 μm (**m**)

To explore the role of DHODH in OSCC development, we examined the impact of DHODH expression on OSCC cell proliferation. In line with the results from clinical samples, DHODH expression in OSCC CAL-27, HSC-3, and HN4 cell lines are much higher than human normal oral keratinocytes (HOK) (Fig. 2a). Since HSC-3 cells showed the highest expression level, we thus knockdown DHODH expression in this cell line by shRNAs (Fig. 2b). As results, loss of DHODH markedly reduced the cell viability by nearly 70%, shown by CCK8 assay and Edu incorporation assay (Fig. 2c–d). Consistently, in colony formation assay, the average number of clones formed by untreated HSC-3 cells was 85.33 ± 3.18, while treatment of shRNA #1 or #2 reduced the clone number to 13 ± 3.46 or 11 ± 3.06, respectively (Fig. 2e). These changes in OSCC cell proliferation were not likely due to the off-target effects, since these two shRNAs targeted distinct sites on DHODH mRNA. Similar results were obtained from HN4 cells (Fig. S1a–c).

Next, we tested whether enzymatic activity is prerequisite for DHODH-dependent OSCC cell proliferation. BAY 2402234 was reported as a selective potent antagonist of DHODH, through binding the ubiquinone binding site of DHODH between the N-terminal helices.^12^ Indeed, incubation with BAY 2402234 obliterate roughly 90% of DHODH activity in the HSC-3 cell lysates (Fig. 2f). Notably, BAY 2402234 treatment markedly abolished the light absorption values in CCK8 assay (Fig. 2g), the percentage of Edu-positive cells in Edu incorporation assay (Fig. 2h), and the clone numbers in colony formation assay (Fig. 2i). Similar results were observed by the treatment with another DHODH inhibitor Vidofludimus (Fig. 2f–i).^13^

To estimate the role of DHODH in regulating OSCC tumor growth, we established a mouse xenograft model by subcutaneous injection of HSC-3 cells that were stably expressed with non-target control (NC) shRNA or DHODH shRNAs. In spite of no obvious differences during the first 8 days after injection, the tumors formed by both DHODH shRNA-expressed cells grew much slower than the NC control tumors (Fig. 2j). Accordingly, the number of cells with Ki-67 expression was markedly decreased upon loss of DHODH (Fig. 2k). Consistently, inhibition of DHODH by BAY 2402234 or Vidofludimus treatment largely retarded xenograft growth and abolished Ki-67-positive cells (Fig. 2l–m). These results suggest that DHODH expression was required for OSCC cell proliferation.

### Inhibition of DHODH impairs pyrimidine nucleotide synthesis

**Fig. 3:**
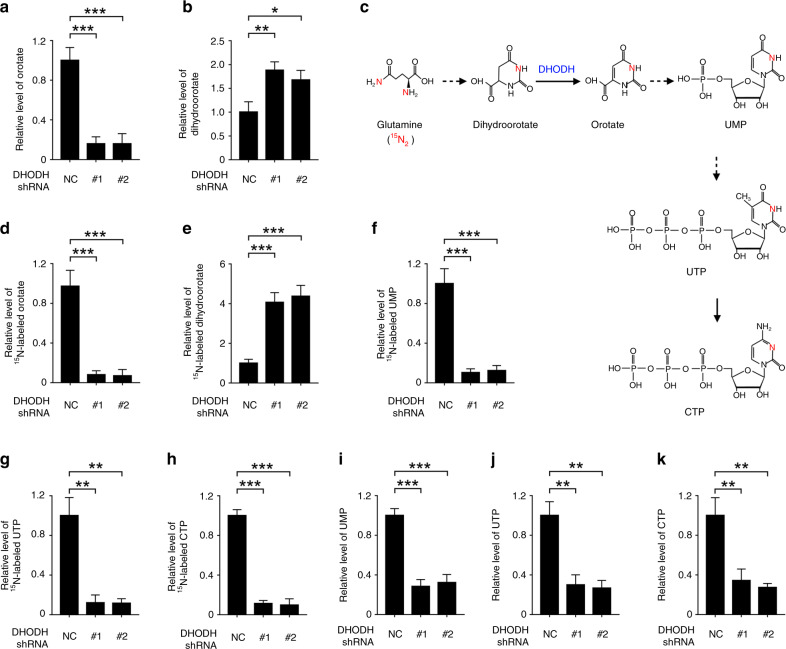
Inhibition of DHODH imped pyrimidine synthesis. **a**, **b**, **i**, **j** HSC-3 cells were transfected with NC or DHODH shRNAs. 48 h after transfection, the indicated metabolite was examined. *P < 0.05; **P < 0.01; ***P < 0.001. **c** A schematic for the transfer of ^15^N in the de novo pyrimidine synthesis. Solid lines indicate one reaction step, and dotted lines indicate multiple reaction steps. **d**–**h** HSC-3 cells were transfected with NC or DHODH shRNAs. 48 h after transfection, cells were incubated with 4 mM ^15^N-labeled glutamine for 1 h. The indicated metabolite was examined. **P < 0.01; ***P < 0.001

Considering that cancer cells frequently hijack nucleotide metabolism to boost cell proliferation,^14^ we examined the role of DHODH in cellular de novo pyrimidine synthesis in OSCC cells. As shown in Fig. 3a, b, loss of DHODH resulted in a compromised level of orotate and an augmented level of dihydroorotate in HSC-3 cells. To further monitor the metabolic flux of this pathway, we incubated HSC-3 cells with ^15^N-labeled glutamine to trace the metabolites generated from glutamine (Fig. 3c). Indeed, shRNA-mediated silence of DHODH substantially eliminated glutamine-derived orotate in HSC-3 cell lysates, with an obvious accumulation of glutamine-derived dihydroorotate, suggesting a blockage in conversion of dihydroorotate to orotate (Fig. 3d, e). Accordingly, the levels of ^15^N-labeled downstream metabolites, including uridine monophosphate (UMP), uridine triphosphate (UTP), and cytidine triphosphate (CTP), were dampened in the DHODH shRNAs-expressed cells (Fig. 3f–h, and S1d).

Salvage pathway support cellular pyrimidine pool by recycling nucleosides and free bases generated by DNA and RNA breakdown, and functions as an alternative route when de novo synthesis pathway is retarded.^15^ However, DHODH-silenced HSC-3 cells exhibited a 60–70% reduction in the level of total cellular UMP, UTP, or CTP, compared to NC shRNA-treated cells (Fig. 3i–k). These data suggest that DHODH is required for pyrimidine nucleotide synthesis in OSCC cells, which cannot be compensated by the salvage pathway.

### Inhibition of DHODH destabilizes mitochondrial contact site and cristae organizing system (MICOS) complex and disrupts mitochondrial homeostasis

**Fig. 4:**
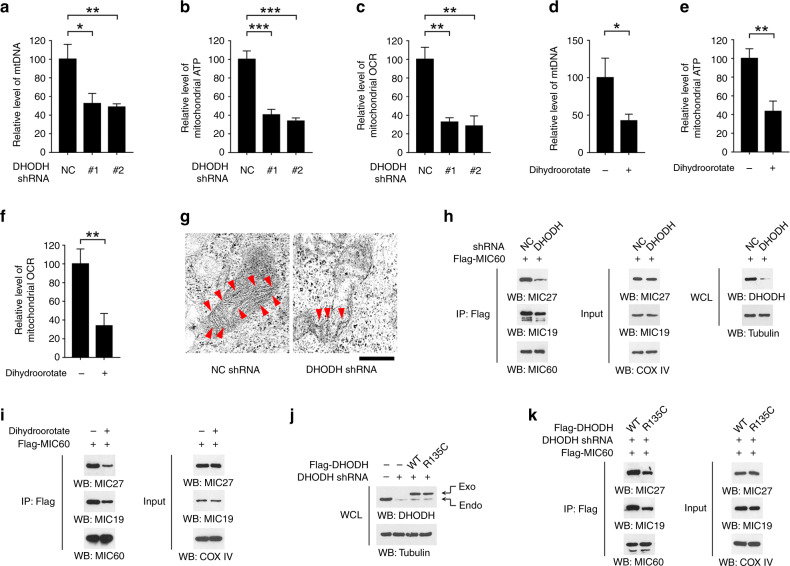
Inhibition of DHODH disrupts mitochondrial homeostasis. **a**, **b**, **c** HSC-3 cells were transfected with NC or DHODH shRNAs. 48 h after transfection, the amounts of mtDNA (**a**), the level of mitochondrial ATP (**b**), and mitochondrial OCR (**c**) were examined. *P < 0.05; **P < 0.01; ***P < 0.001. **d**–**f** HSC-3 cells were incubated with 5 μM dihydroorotate for 4 h, the amounts of mtDNA (**d**), the level of mitochondrial ATP (**e**), and mitochondrial OCR (**f**) were examined. *P < 0.05; **P < 0.01; ***P < 0.001. **g** HSC-3 cells were transfected with NC or DHODH shRNAs. 48 h after transfection, the ultrastructure of mitochondria was examined by TEM. Arrowhead indicates cristae. Scale bar, 500 nm. **h** HSC-3 cells were transfected with NC or DHODH shRNAs. 48 h after transfection, mitochondria was isolated and immunoprecipitation was performed using indicated antibodies. **i** HSC-3 cells were incubated with 5 μM dihydroorotate for 4 h. Mitochondria was isolated and immunoprecipitation was performed using indicated antibodies. **j**, **k** HSC-3 cells were transfected with DHODH shRNAs, WT Flag-DHODH, or Flag-DHODH R135C mutant (**j**). 48 h after transfection, mitochondria was isolated and immunoprecipitation was performed using indicated antibodies (**k**)

DHODH protein is mainly located in the mitochondrial inner membrane.^11^ Strikingly, the amount of mitochondrial (mt)DNA was largely reduced in DHODH-silenced HSC-3 cells (Fig. 4a). Therefore, we examined the potential involvement of DHODH in mitochondrial homeostasis. As shown in Fig. 4b–c, knockdown of DHODH diminished both the ATP content and oxygen consumption rate (OCR) in mitochondria isolated from HSC-3 cells. As normalized to the total mass of mitochondrial protein, the lowered mitochondrial ATP and OCR was supposedly due to mitochondrial dysfunction, rather than the reduced amounts of mitochondria. Additionally, since loss of DHODH caused accumulation of cellular dihydroorotate (Fig. 3b), we hypothesized that the treatment effect of dihydroorotate might recapitulate the effects of DHODH shRNA. Indeed, incubation with dihydroorotate resulted in an obliterate mtDNA as well as a repressed mitochondrial ATP and OCR (Fig. 4d–f).

Mitochondrial ultrastructure analyses by transmission electron microscopy (TEM) revealed a reduced and disturbed structure of mitochondrial cristae in DHODH-silenced cells (Fig. 4g). Mitochondrial cristae are folds of the inner membrane, which provides the indispensable platform for respiratory chain.^16^ MISCO complex, formed by a set of MIC protein components, plays a pivotal role in maintaining the proper structure of mitochondrial cristae.^17^ Of our particular interest, we tested the impact of DHODH on MISCO complex assembly. We found that either knockdown of DHODH (Fig. 4h and S1e) or treatment with dihydroorotate (Fig. 4i) partially abolished the amount of MIC27, MIC19, or MIC10 proteins in MIC60 immunoprecipitates, suggesting that loss of DHODH may destabilize the MISCO complex. Further, we knockdown the endogenous DHODH in HSC-3 cells and reconstituted the expression of an enzymatic-dead R135C mutant,^18^ and found that this mutant similarly attenuated the interactions between MIC protein components (Fig. 4j–k), suggesting the enzymatic activity of DHODH was required for maintaining MISCO complex. Together, these results suggest that inhibition of DHODH causes mitochondrial dysfunction probably through disturbing MISCO complex assembly.

### Sex determining region Y-box 2 (SOX2) transcriptionally regulates DHODH expression in OSCC

**Fig. 5:**
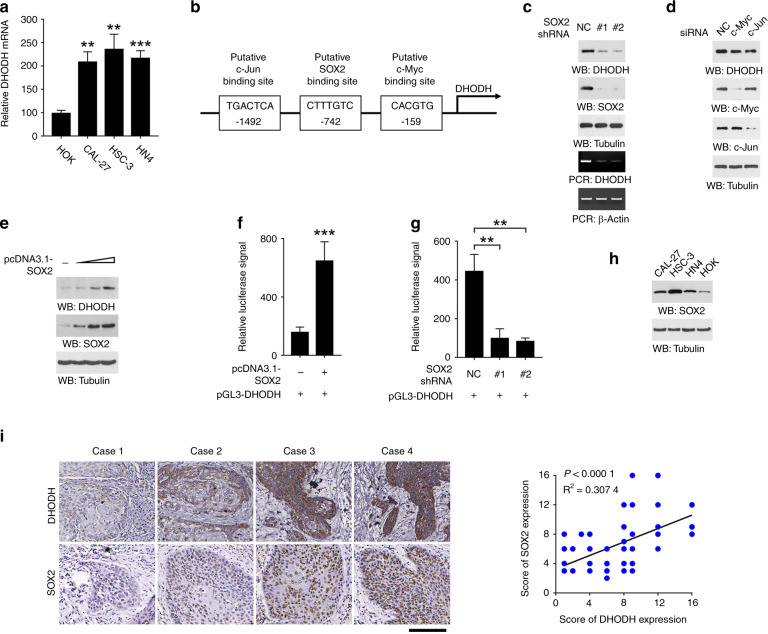
SOX2 regulates DHODH expression in OSCC cells. **a** Expression of DHODH in the indicated cell lines were examined by RT-PCR. **b** A schematic for the putative binding sites of c-Jun, SOX2, or c-Myc in the promoter region of DHODH. **c**, **d**, **g** HSC-3 cells were transfected with SOX2 shRNA (**c**), c-Myc siRNA, or c-Jun siRNA (**d**). 48 h after transfection, expression of DHODH was examined by immunoblot or RT-PCR (**c**, **d**). DHODH transcription activity was examined by luciferase assay (**g**). **e**–**f** HOK cells were transfected with SOX2 expressing vector. 48 h after transfection, expression of DHODH was examined by immunoblot (**e**). DHODH transcription activity was examined by luciferase assay (**f**). **h** Expression of SOX2 in the indicated cell lines were examined by immunoblot. **i** Correlation between DHODH and SOX2 expression was analyzed. Note that the dots representing those cases with the same SOX2 and DHODH staining score were overlapped. Scale bar, 150 μm

Next, we turned to explore the mechanism responsible for DHODH upregulation in OSCC. Quantification of DHODH mRNA showed an apparent increased in CAL-27, HSC-3, and HN4 cells compared to HOK cells (Fig. 5a), elucidating that DHODH was likely regulated at transcriptional level. Analyses of DHODH promoter region (−1500 – 0) by PROMO online engine (Version 8.3, http://alggen.lsi.upc.es/cgi-bin/promo_v3/promo/promoinit.cgi?dirDB=TF_8.3) revealed putative binding sites for three OSCC-associated transcriptional factors, c-Jun (ref. ^19^), SOX2 (ref. ^20^), and c-Myc (ref. ^21^) (Fig. 5b). However, only knockdown of SOX2 largely abolished DHODH expression in HSC-3 cells (Fig. 5c–d). Further, transfection of various amounts of DHODH-expressing vector caused an enhanced DHODH expression at a distinct extent in HOK cells (Fig. 5e), suggesting that SOX2, but not c-Jun or c-Myc, was involved in the regulation of DHODH transcription. Consistently, modulation of SOX2 expression in HOK (Fig. 5f) or HSC-3 cells (Fig. 5g) efficiently affected the transcriptional activity of DHODH, shown by a luciferase-based reporter gene assay.

We determined the correlation between the expression of DHODH and SOX2. As shown in Fig. 5h, the expression pattern of SOX2 among HOK and OSCC cell lines was comparable to DHODH expression. Further, immunostaining of SOX2 revealed that most cases with high DHODH expression showed a stronger SOX2 immunoreactivity (Fig. 5i). Quantification of the staining showed that these correlations were significant. Taken together, these results suggest that upregulation of DHODH in OSCC is regulated by SOX2 at transcriptional level.

## Discussion

Nucleotide metabolism fuel DNA replication and RNA synthesis, which is indispensable for cell proliferation. But how tumor cells orchestrate nucleotide metabolic enzymes to support their rapid growth is largely unknown.^22^ Here, we show that pyrimidine metabolic enzyme DHODH was overexpressed in OSCC, and is correlated with poor outcome of OSCC patients. Further, inhibition of DHODH by either shRNA or selective inhibitor counteracted OSCC cell proliferation and xenograft growth. Our data suggest that DHODH might be a prognostic biomarker or potential treatment target for OSCC.

We demonstrate that loss of DHODH blocked pyrimidine synthesis, and abolished cellular pyrimidine pool, which was unlikely replenished by salvage pathway. Tumor cells frequently harbor an enhanced de novo synthesis of pyrimidine nucleotides to support the rapid cell proliferation.^23^ Pyrimidine nucleotides are essential building blocks for DNA replication and RNA transcription.^24^ Additionally, CTP is prerequisite for phosphatidylcholine synthesis, which is the major component of cell membrane.^25^ While UTP has a role in the metabolism of galactose, where the activated form UDP-galactose is converted to UDP-glucose.^26^ Considering DHODH governs the rate-limiting step for pyrimidine synthesis, it is reasonable to infer that inhibition of DHODH impairs OSCC cell proliferation by disrupting pyrimidine nucleotide supply.

It was an unexpected finding that knockdown of DHODH defaced MICOS complex and perturbed the cristae structure in mitochondria. Mitochondria are essential organelles within all proliferating cells, including tumor cells. Mitochondria are responsible for nutrients transport, energy metabolism, and synthesis of numerous cellular components required for growth and proliferation.^24^ These functions of mitochondria are largely dependent on cristae formed by the folding of the inner membrane, whose structure increases the surface area of the inner membrane. MICOS protein complexes are crucial for the formation and maintenance of the cristae structure.^16^ Nine types of MICOS complex subunits have been described so far, including MIC 60 (mitofilin), MIC 10 (MINOS1), MIC 19 (CHCHD3), MIC 25 (CHCHD6), MIC 26 (APOO), MIC 23, MIC 27 (ApooL), MIC 13 (Qil1), and MIC 14 (CHCHD10). Accumulating evidences indicate that MIC60 is the core component of MICOS. MIC 60, mitochondrial outer membrane protein Sam 50 and MIC 19 interact with each other to form bridge-like complex, which mediates the contact between the mitochondrial outer and inner membranes.^27,28^ Considering that mitochondrial respiration complexes are assembled and located on the inner membrane, it is reasonable to infer that impaired mitochondrial respiration, observed in DHODH-silenced cells, was probably caused by the modulation of the MICOS complex. In addition, since we also show that treatment with dihydroorotate caused similar effects on mitochondria, further work is still needed to decipher the biological consequences for dihydroorotate overload in mitochondrial inner membrane.

Further, our data suggest that SOX2 probably governs DHODH expression in OSCC. We show that both expression or transcription activity of DHODH in OSCC cells could be manipulated by modulating the expression SOX2. SOX2 plays an important role in the regulation of embryonic cell development and the stem-cell nature of various adult stem cell populations.^29^ However, SOX2 is frequently activated in tumor cells with stem cell-like characteristics, regulating cell self-renewal, maintaining cell pluripotency, and thereby promoting tumor growth. The abnormal expression of SOX2, probably due to increased copy number in genome, is associated with multiple tumor types, including OSCC.^30^ It is reported that upregulation of SOX2 is associated with a more aggressive phenotype of OSCC cells, and rendered OSCC cells resistant to chemotherapy.^31^ In this study, our data underscores the role of SOX2 in the reprogramming of pyrimidine synthesis, through depicting its regulation of DHODH transcription. More work is merited to determine whether SOX2 also regulates other steps of nucleotide synthesis, such as the pentose phosphate pathway or salvage pathway.

## Materials and Methods

Antibodies recognizing Tubulin, COX IV, SOX2, c-Myc, were obtained from Cell Signaling Technology. Antibodies recognizing DHODH, and MIC19 were obtained from Abcam. Antibody against Flag, anti-Flag M2 agarose beads, dihydroorotate, and bovine serum albumin were purchased from Sigma. Antibody recognizing MIC60 was obtained from NOVUS. Antibody recognizing MIC10 was obtained from PROSPEC. Antibody recognizing MIC27, horseradish peroxidase-conjugated goat anti-mouse, or rabbit secondary antibodies were obtained from Thermo Fisher Scientific. [^15^N_2_]-glutamine was purchased from Cambridge Isotope Laboratories. BAY 2402234 and Vidofludimus were obtained from TOPSCIENCE. Lipofectamine 2000 was obtained from Thermo Fisher Scientific.

### DNA constructs and mutagenesis

PCR-amplified human DHODH, MIC60, SOX2, and DHODH promoter region (−1 500-0) were subcloned into pcDNA3.1/hygro(+)-Flag, or pGL3 vectors. DHODH R135C mutant was constructed using the QuikChange site-directed mutagenesis kit (Stratagene, La Jolla, CA).

The following pGIPZ shRNAs were used: control shRNA, GCT TCT AAC ACC GGA GGT CTT; DHODH shRNA #1, AAT ATT CAA TGT CCT TGC A (targeting non-coding region); DHODH shRNA #2, TCC ATG ACT TTT TCC TCC T (targeting non-coding region); SOX2 shRNA #1, TCT CAG CTT ATA AAC AAT G; SOX2 shRNA #2, AAA ACA TTT TTT TCG TCG C. Mixed siRNAs targeting c-Myc or c-Jun was obtained from Santa Cruz.

### Cell culture

CAL-27 cells were obtained from ATCC, and HSC-3 cells were obtained from JCRB Cell Bank. HOK cells were obtained from ScienCell. HN4 cells were provided by J. Silvio Gutkind. These cells maintained with Dulbecco’s Modified Eagle Medium supplemented with 10% fetal bovine serum. Cells were treated with BAY 2402234 at 10 nM or Vidofludimus at 10 μmol·L^−1^.

For generating shRNA-depleted stable cell lines, cells were transfected with shRNA plasmids and selected by puromycin. For generating gene-expressing stable cell lines, shRNA-depleted cells were infected with lentivirus carrying WT or mutant gene and selected by hygromycin or/and G418.

Transfection of plasmids were performed by using Lipofectamine 2000, following the manufacture’s instructions.

### Immunoprecipitation and immunoblot analysis

Protein was extracted from cultured cells using a lysis buffer (50 mmol·L^−1^ Tris-HCl [pH 7.5], 0.1% SDS, 1% Triton X-100, 150 mmol·L^−1^ NaCl, 1 mmol·L^−1^ dithiothreitol, 0.5 mmol·L^−1^ EDTA, 100 μmol·L^−1^ PMSF, 100 μmol·L^−1^ leupeptin, 1 μmol·L^−1^ aprotinin, 100 μmol·L^−1^ sodium orthovanadate, 100 μmol·L^−1^ sodium pyrophosphate, and 1 mmol·L^−1^ sodium fluoride). The cell lysates was centrifuged at 13 400 g, and supernatants (2 mg protein per mL) were immunoprecipitated overnight at 4 °C using the indicated antibodies. Following the overnight incubation, protein A or G agarose beads were added and left for an additional 3 h. The immunocomplexes were then washed with lysis buffer 3 times and prepared for immunoblot analyses with corresponding antibodies as described previously.

### Analysis of intermediate metabolites

Metabolites were confirmed by high-resolution mass spectrometry (HRMS). For preparation of cell sample, 5 × 10^7^ cells were washed with ice-cold PBS to remove culture medium. The tissue samples (500 mg) of mice intestine were homogenized on ice immediately after sampling. Metabolites were extracted by treatment with 90/9/1 (v/v/v) acetonitrile/water/formic acid. The samples were centrifuged at 117 000 g for 10 min, and then the supernatant was transferred to a clean tube and evaporated to dryness under a nitrogen gas stream. Dried samples were reconstituted in 0.2% ammonium hydroxide in ammonium acetate (10 mmol·L^−1^), then 10 μL was injected into a Thermo Scientific Vanquish liquid chromatography system containing a Thermo Hypercarb 100 × 3 mm 3 μm HPLC column heated to 35 °C with mobile phase A (MPA) consisting of 0.2% ammonium hydroxide in ammonium acetate (10 mmol·L^−1^) and mobile phase B (MPB) consisting of 0.2% ammonium hydroxide in acetonitrile. The gradient elution was performed at a flow rate of 0.3 mL·min^−1^: 0 min (0% MPB)-2.0 min (0% MPB)-15.0 min (30% MPB)-15.1 min (95% MPB)-20.0 min (95% MPB)-20.1 min (0% MPB)-25.0 min (STOP). Thermo Orbitrap Fusion Tribrid Mass Spectrometer in Selected Ion Mode (SIM) electrospray positive mode was used to acquire data. Then, use Thermo TraceFinder software to qualitatively analyze the Peak integration and area.

Cells were incubated with [^15^N_2_]-glucose (4 mmol·L^−1^), for indicated time to track metabolic flux. The metabolites were analyzed by HRMS with similar protocol.

### EdU incorporation assay

EdU incorporation assay was performed by using EdU Proliferation Kit (Abcam). Briefly, after treatment, cells were incubated with 20 µmol·L^−1^ EdU for 3 h. Cells were then sequentially incubated with 1X Fixative Solution for 15 min, 1X Permeabilization Buffer for 20 min, EdU Additive Solution Reaction Buffer for 30 min, and 5 µg·mL^−1^ Hoechst for 30 min. The EdU- or Hoechst-positive cells were counted in at least 20 fields. The percentage of Edu-positive cells was calculated as the ratio between EdU- and Hoechst-positive cells.

### Colony formation assay

Cells were seeded into a six-well plate at a density of 100 cells per well, and cultured for 14 days. Colonies were fixed and stained with Crystal Violet solution (0.5% Crystal violet in 20% ethanol), colonies of more than 50 cells were counted.

### CCK8 assay

Cell Counting Kit 8 (WST-8/CCK8) (Abcam) assessed Cell proliferation. Cells were seeded in a 96 well plate (5 000 cells per well) overnight. A total of 10 µL of the cell proliferation reagent WST-8 solution was added to each well of the 96-well plate and then incubated for 3 h at 37 °C in dark. The change in absorbance was measured at 460 nm.

### Immunohistochemical staining

Immunohistochemical staining was performed according to manufacturer instructions using the VECTASTAIN ABC kit (VECTOR LABORATORIES, CA). Sections were scored according to the percentage of positive cells and staining intensity. Scoring was established as follows: the percentage of positive cells was scored as 0 if 0% of the tumor cells showed positive staining, 1 if 0% to 10%, 2 if 11% to 30%, 3 if 31% to 70%, and 4 if 71% to 100%, the staining intensity was rated on a scale of 0 to 4: 0, negative; 1, weak; 2, moderate; 3, strong; 4, very strong. We then multiply the proportion and intensity scores to obtain a total score (range: 0–16).

### Transmission electron microscopy (TEM)

The cells were fixed with 0.1% glutaraldehyde in 0.1 mol·L^−1^ sodium iodate and 1% OsO_4_ successively for 2 h and 1.5 h, respectively. Then the samples were stained in 3% uranyl acetate aqueous solution for 1 h after washing. After that, the samples were rinsed again with water, then dehydrated with gradient alcohol (50%, 75%, and 95%–100% alcohol), and finally embedded in Epon-Araldite resin (Canemco, 034). Ultrathin sections were prepared on Reichert Ultramicrotome, and the sections were counterstained with 0.3% lead citrate, and then observed under a Philips EM420 transmission electron microscope. Use Image Pro Plus version 3 to obtain the value of the area occupied by autophagic vesicles and cytoplasm.

### Reverse transcription polymerase chain reaction (RT-PCR)

RT-PCR was performed following a previous report.^32^ The primers are as follows: DHODH forward, GCT GTC ATT AAC AGG TAT GGA TTT AAC AG; DHODH reverse, GTT GAT AAA TCC CGG AGG GGC TTC.

### Measurement of mitochondrial DNA content

QIAamp DNA mini kit (QIAGENE, Germantown, MD) was used to extract total DNA according to the manufacturer’s instructions. The level of mitochondrial DNA D-loop structure was measured by qRT-PCR and standardized to β-actin encoded by genomic DNA. The primers are as follows: D-Loop forward, GAT TTG GGT ACC ACC CAA GTA TTG; D-Loop reverse, GTA CAA TAT TCA TGG TGG CTG GCA; Actin forward, TCA CCC ACA CTG TGC CCA TCT ACG A; Actin reverse, CAG CGG AAC CGC TCA TTG CCA ATG G.

### Measurement of DHODH activity

DHODH activity in cell lysates were measured following the previous report.^33^ A total of 300 μL cell lysate was mixed with in an aqueous solution (total volume, 1.0 mL) containing 500 μmol·L^−1^ DHO, 200 mmol·L^−1^ K_2_CO_3_-HCI (pH 8.0), 0.2% triton X-100, and 100 μmol·L^−1^ coenzyme Q10 at 37 °C 30 min. An aliquot (100 μL) of the mixture of enzyme reaction mixture was mixed with 100 μL of 0.5 μmol·L^−1^orotic acid, 50 μL of H_2_O, 250 μL of 4.0 mmol·L^−1^ 4-TFMBAO, 250 μL of 8.0 mmol·L^−1^ K_3_[Fe(CN)_6_], and 250 μL of 80 mmol·L^−1^ K_2_CO_3_ and then heated at 80 °C for 4.0 min. The reaction was stopped by cooling in an ice-water bath and the FL intensity was measured with a spectrofluorometer (FP-6300 Jasco, Tokyo, Japan): excitation and emission wavelengths were 340 nm and 460 nm, respectively.

### Mitochondria isolation

Mitochondria was isolated by using Mammalian Mitochondria Isolation Kit (Biovision), following the manufacture’s instruction.

### Measurement of ATP content

Mitochondrial ATP content was measured in freshly isolated mitochondrial by using ATP Assay Kit (Colorimetric/Fluorometric, Abcam), following the manufacture’s instruction.

### Measurement of mitochondrial OCR

Mitochondrial OCR was measured in freshly isolated mitochondrial by using MitoCheck® Mitochondrial OCR Assay Kit, following the manufacture’s instruction.

### Mice xenograft

A total of 1 × 10^6^ HSC-3 cells was subcutaneously implanted into 6-week-old female athymic nude mice (*n* = 7). The tumor volume was calculated by the formula: 0.5 × length × width^2^. The mice were sacrificed 27 days after implantation, and tumor tissues were subjected to immunohistochemical staining.

### Quantification and statistical analysis

Statistical analysis was performed using the two-tailed unpaired Student’s t-test with a *P*-value of < 0.05 was considered as statistical significance unless specifically indicated. All data were analyzed as the mean ± standard deviation from three independent experiments/samples unless otherwise stated. Significance levels are: *P < 0.05; **P < 0.01; ***P < 0.001.

## Supplementary information

Suppl figure S1

Suppl figure legend
