# Supplementary figures and images for: SOX2-dependent expression of dihydroorotate dehydrogenase regulates oral squamous cell carcinoma cell proliferation
Source: Int J Oral Sci. 2021 Jan 29;13:3. doi: 10.1038/s41368-020-00109-x (PMC7844284; doi:10.1038/s41368-020-00109-x)

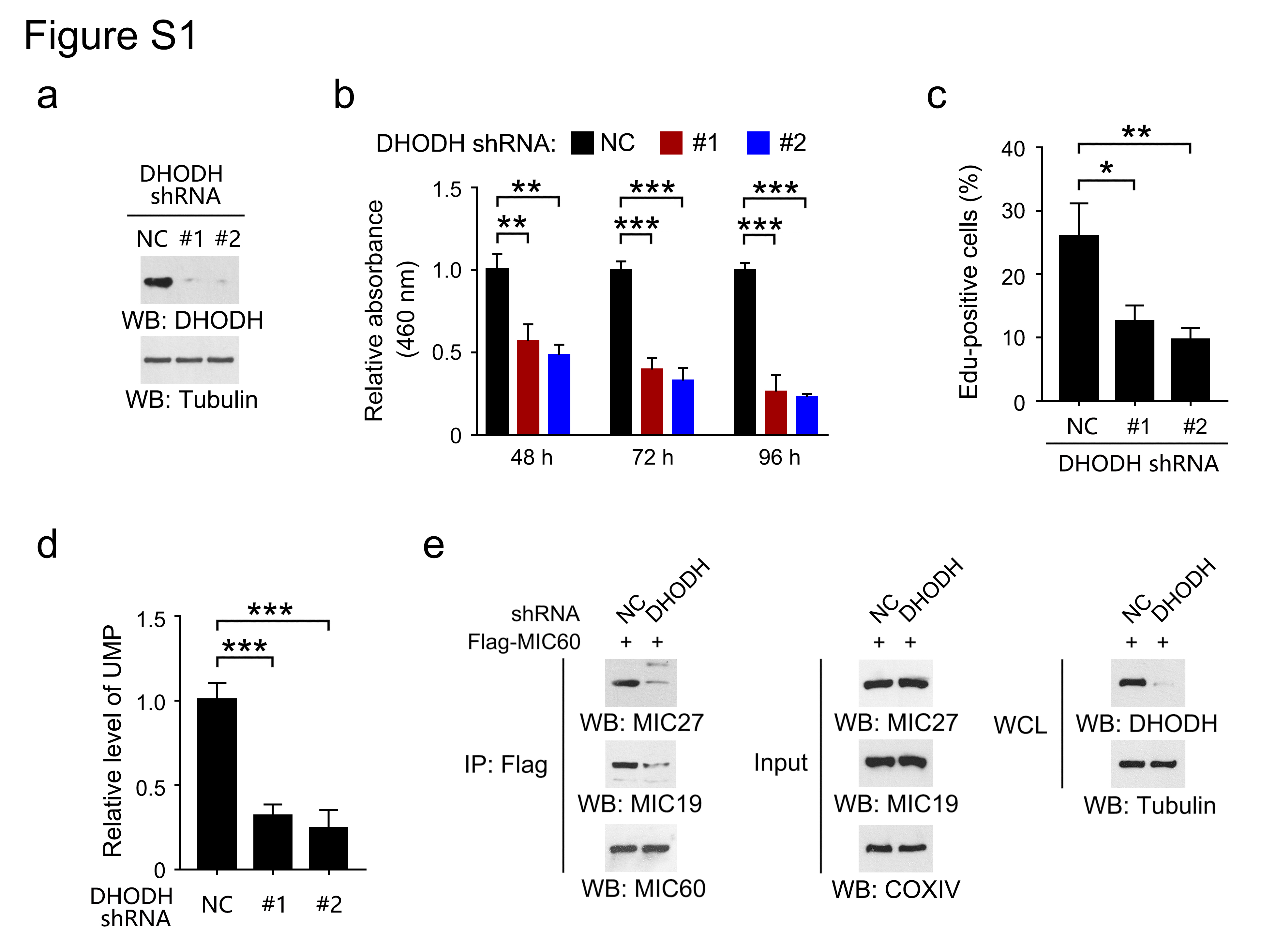

Supplement: Supplementary file 1 — Suppl figure S1 [file 41368_2020_109_MOESM1_ESM.jpg]
