# Supplementary material for: SOX2-dependent expression of dihydroorotate dehydrogenase regulates oral squamous cell carcinoma cell proliferation
Source: Int J Oral Sci. 2021 Jan 29;13:3. doi: 10.1038/s41368-020-00109-x (PMC7844284; doi:10.1038/s41368-020-00109-x)
Supplement: Supplementary file 2 — Suppl figure legend [file 41368_2020_109_MOESM2_ESM.pdf]

Supplementary Figure legends

Figure S1

(a) HN4 cells were transfected with NC or DHODH shRNA. 48 h after transfection, the expression of DHODH was examined by immunoblot.

(b) HN4 cells were transfected with NC or DHODH shRNA. 48, 72 or 96 h after transfection, cell viability was examined by CCK-8 assay. Data were normalized to the NC shRNA-treated group of the same time point. **, $P < 0.01$; ***, $P < 0.001$.

(c) HN4 cells were transfected with NC or DHODH shRNA. 72 h after transfection, cell proliferation was examined by EdU incorporation assay. *, $P < 0.05$; **, $P < 0.01$.

(d) HN4 cells were transfected with NC or DHODH shRNA. 48 h after transfection, the level of UMP was examined. ***, $P < 0.001$.

(e) HN4 cells were transfected with NC or DHODH shRNA. 48 h after transfection, mitochondria was isolated and immunoprecipitation was performed using indicated antibodies.
